# Hyperostosis Fronto-Parieto-Occipitalis: A Cadaveric Case Report

**DOI:** 10.7759/cureus.41445

**Published:** 2023-07-06

**Authors:** Emily Otken, Emily O'Brien, Braden Nyboer, Huy Nguyen, Cody Orvin, Adegbenro O Fakoya

**Affiliations:** 1 Anatomy, Louisiana State University Health Sciences Center, Shreveport, USA; 2 Cellular Biology and Anatomy, Louisiana State University Health Sciences Center, Shreveport, USA

**Keywords:** morgagni–stewart–morel syndrome, troell–junet syndrome, hyperostosis frontalis interna, hyperostosis cranialis interna, hyperostosis

## Abstract

Hyperostosis of the skull is a rare bone dysplasia described in disorders such as hyperostosis cranialis interna (HCI) and hyperostosis frontalis interna (HFI). Other syndromes presenting with hyperostosis include Morgagni-Stewart-Morel (MSM) and Troell-Junet. HCI is an abnormal hyperostosis of most endosteal skull and calvarium surface regions. A more specific hyperostosis, HFI, is an unusual bone growth based on its volume and porosity; it is primarily located bilaterally on the frontal portions of the calvarium. However, the hyperossification does not cross the superior sagittal sinus. Upon cadaveric dissection, we found hyperossification beyond the frontal area, extending to the parietal and occipital bones with the significant characteristic of no midline interference. Hyperossification results in gross indentations on the corresponding frontal, parietal, and occipital hemispheric brain tissues. This report discusses possible differentials for this rare cadaveric finding of frontal, parietal, and occipital bone hyperostosis. This case report includes some major characteristic features indicative of HCI and HFI with some interesting variations and features suggestive of MSM and Troell-Junet syndromes. Due to the lack of patient history and medical records, no further conclusions about clinical differentials, symptoms, or causative syndromes could be drawn; further research needs to be conducted on HCI, HFI, and related syndromes to understand their presentations better.

## Introduction

Hyperostosis of the skull is a rare bone dysplasia that is described in many different disorders. There is ongoing research on this topic, and many questions still need to be answered. Hyperostosis cranialis interna (HCI) is a unique bone disorder characterized by irregular bone growths on the skull base or calvaria. It is a rare hereditary disease reported only in a few European families. Patients present with various symptoms, from asymptomatic to mild/severe brain deficits [1].

Another similar disease, more commonly seen in many case reports, is hyperostosis frontalis interna (HFI) which consists of bone growth only in the frontal part of the skull. However, this disease can be classified depending on the appearance, shape, size, and involvement of other bones. Type A-D involves increasing degrees of involvement ranging from <25%, 25%, 50%, and >50%, respectively. Dysplasia can sometimes spread from the frontal bone to the parietal and occipital bones. There has been very little research on parietal and occipital involvement, so their relation to one another is not certain. Not much is known about this condition since most patients present with no symptoms, and the only way to see abnormal bone growth is through medical imaging, such as MRI, CT, and X-rays. However, one defining feature in many cases is the absence of bone growth along the superior sagittal sinus. HFI is typically seen in elderly postmenopausal females but case reports show an increased prevalence in obese individuals [2].

The symptoms and severity of HFI and HCI tend to increase with age. The continual bone growth compresses against the brain, decreasing the arterial supply to the respective area. This can cause many potential deficits that vary in significance depending on the affected location. Growth in the frontal bone can cause deficits in Broca’s speech area, the premotor cortex, and the prefrontal association cortex. Growth in the parietal bone can cause deficits in the somatosensory cortex. Likewise, growth in the occipital bone can cause visual deficits [3].

In this case report, we present a male cadaver with hyperostosis of the internal calvarium and discuss possible differentials and symptoms of this condition. This case is unique because reported cases of HFI are usually limited to the frontal bone (hence the name frontalis) and, on a few occasions, the parietal. Here, we present a cadaveric hyperostosis with expansions to the occipital bone.

## Case presentation

**Figure 1:**
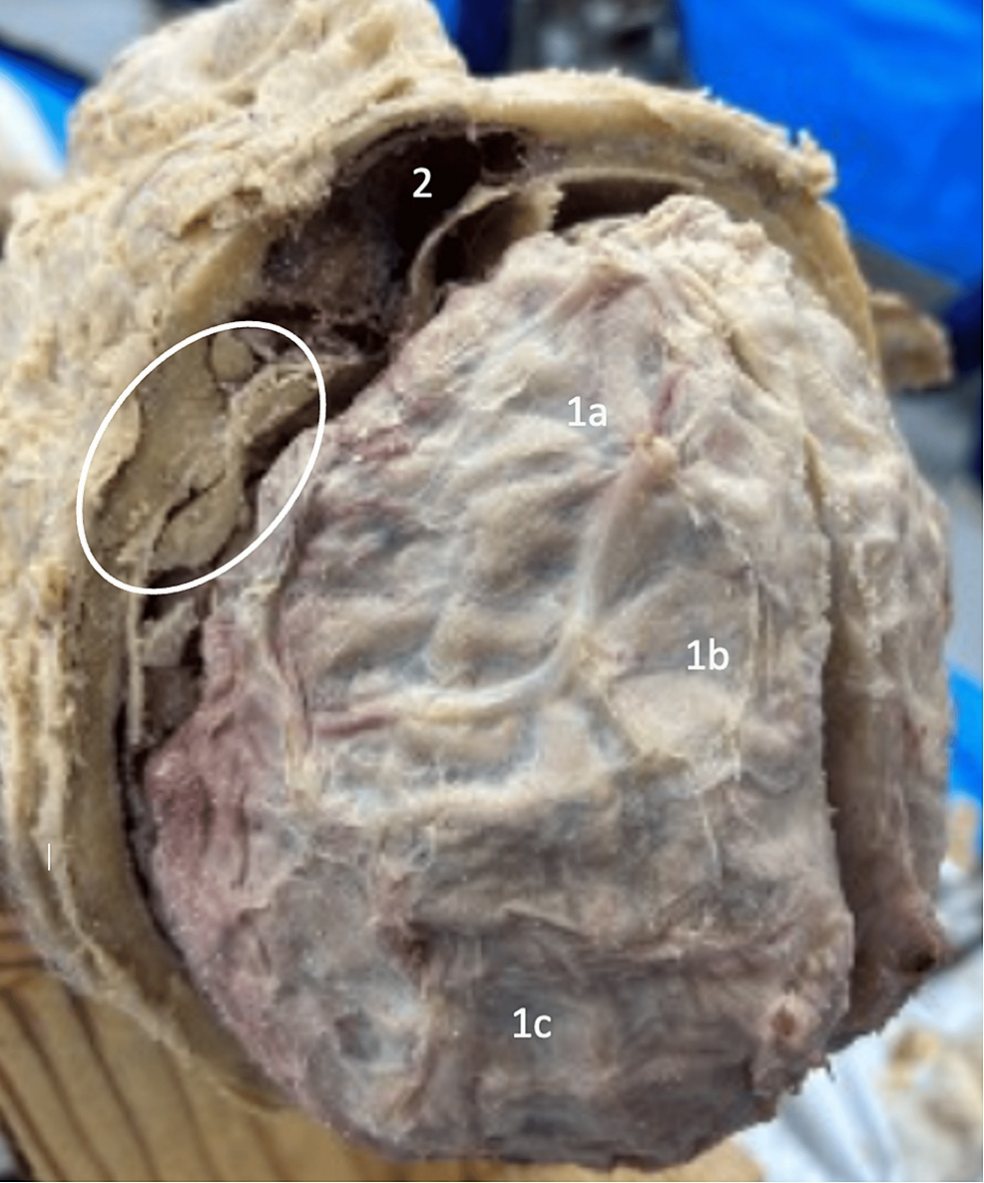
Superior view of the brain Superior view of the brain within the cranial cavity and a removed calvarium showing the corresponding indentations - the porous, bony calvarium placed upon the adjacent brain tissue in the frontal (1a), parietal (1b), and occipital (1c) lobes with a clear view of the posterior-inferior portion of the left frontal sinus within the remaining frontal bone (2). The area encircled represents an osseous expansion from the frontal bone region of the skull.

**Figure 2:**
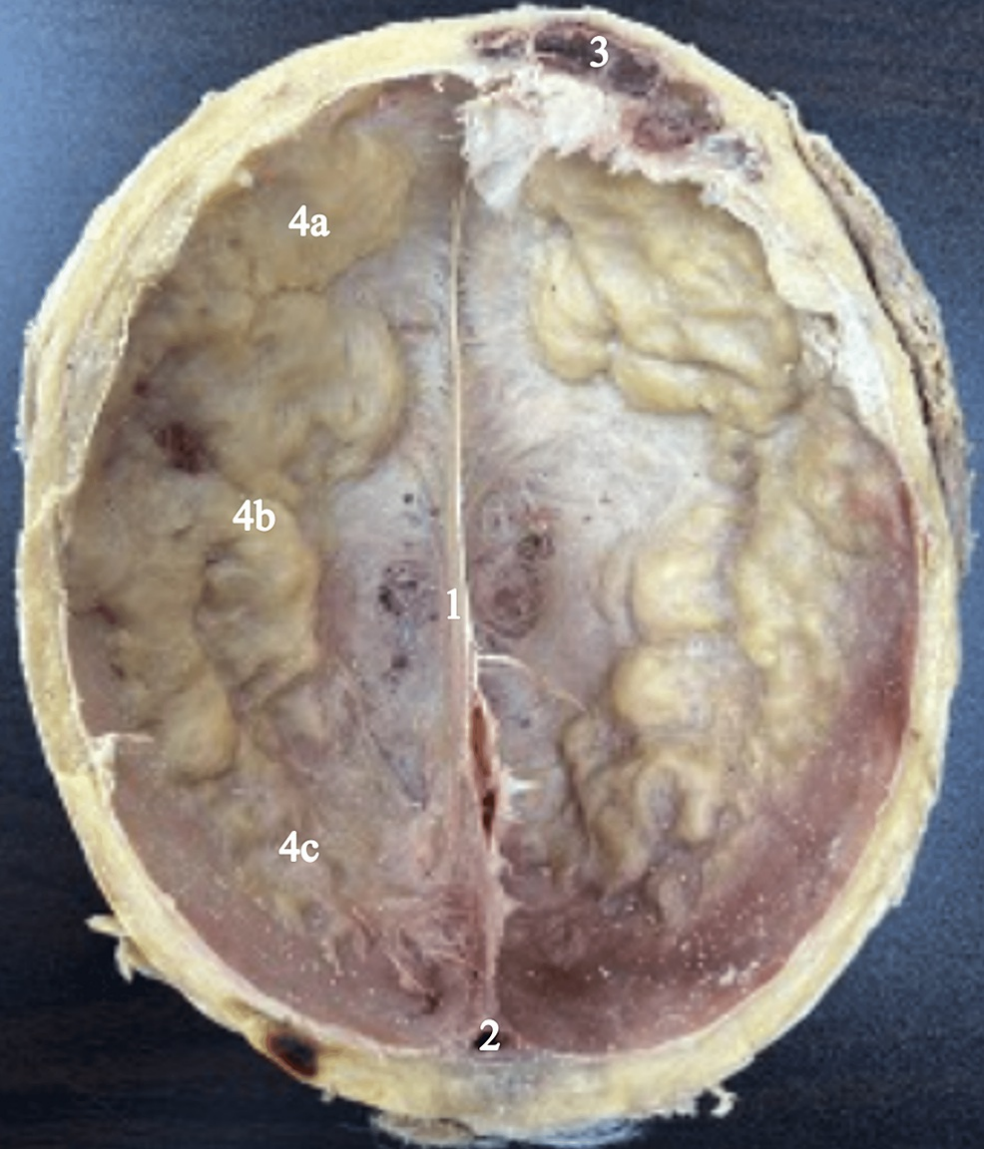
Inferior view of the internal calvarium Inferior view of the internal calvarium with a clear remnant of the falx cerebri (1), confluence of sinus (2), superior portion of left frontal sinus in frontal bone (3), and a thickened porous-appearing bone substance ranging across the frontal (4a), parietal (4b), and occipital (4c) lobes of the periosteum bilaterally.

During the dissection of the skull of an elderly, slightly overweight Caucasian male, we noticed that the superficial skull and overlying tissue possessed no gross abnormalities. Following the removal of the superior portion of the skull, we discovered a thickened bone material ranging across the frontal, parietal, and occipital bones. Upon observation of the internal calvarium, numerous porous-like cavities expanded from the periosteum ranging in size. Given that these porous-like cavities expanded from the frontal, parietal, and occipital lobes, corresponding indentations were also noted within the adjacent brain tissue in the skull (Figure 1). Even though both hemispheres of the calvarium were affected, the hyperostosis did not cross the superior sagittal sinus at any point within the skull. The extent of hyperostosis lesions also increased in size and amount bilaterally in the posterior to the anterior direction. Thus, the frontal lobe appeared to possess the most elevated and substantial amount of hyperostosis reactions within the skull, which did appear to impact and consequently obstruct the left frontal sinus (Figure 2).

## Discussion

Hyperostosis is an abnormality of the bone resulting from excess growth or thickening beyond average bone growth. The most comprehensive form of cranial hyperostosis is HCI. Clinically, hyperostosis is most commonly described within the context of HFI, a thickening of the frontal bone of the calvarium. Case reports regarding HFI also note expansion into the parietal bones, referred to as hyperostosis fronto-parietalis [4]. Our case report reveals the expansion of hyperostosis into the occipital region; we report a rare cadaveric case of hyperostosis fronto-parieto-occipitalis. Due to the cadaveric nature of our study, conclusions as to the cause of hyperostosis in this cadaver cannot be ascertained. HCI is a possible differential, but this diagnosis cannot be confirmed without genetic testing. Other differentials include HFI, MSM, and Troell-Junet syndromes.

HCI presents with hyperostosis of the frontal, parietal, temporal, and occipital bones. Research on HCI has included human clinical studies and animal experimentation to determine the genetic origins of this presentation. Several case presentations noted that the number of families with this disorder is limited. The families were all of Dutch origin, with one report stating that a common ancestor was found among the families [5]. HCI was found to be autosomal dominant in inheritance [1]. Patients with HCI may start showing symptoms within the second decade of life. However, many patients with HFI will show no symptoms at all [1]. HCI patients have severe cranial nerve deficits including sensory, motor, visual, and auditory impairments [1]. The study showed radiographic changes in the skull beginning in the second half of the first decade [1]. Our case best mimics the radiographic finding of HCI, but since ours is a cadaver study, clinical manifestations could not be accounted for. No noticeable cranial nerve compression or abnormalities were recognized during the dissection of the head and neck regions. A comprehensive genetic panel for the region between D8S282 and D8S382 would need to be performed including a genealogical research on the ancestry of our patient [5].

HFI is hyperossification of the frontal bone of the skull. An extensive literature search shows that HFI does not have a definitive genetic origin despite the genetic origin of HCI, therefore concluding that HCI and HFI are unique diagnoses. Research into parietal bone ossification was discussed in the literature as a variation of HFI. Therefore, this section discusses associations of HFI as a whole.

HFI has unequal distribution among diagnosed genders. Raikos et al. investigated the frequency of HFI among cadaver and dry skull samples from male and female specimens [6]. The study also analyzed multiple age groups to determine when hyperostosis presents in an individual. They concluded that HFI is much more common in females than males and in people over the age of 65 [6]. Our study is unique in that our specimen was an elderly male. All known cases of HFI in males have been associated with diseases, syndromes, or treatments causing androgen deficiency, such as testicular atrophy, Klinefelter syndrome, and Kallman syndrome [7]. Our subject had bilateral indirect inguinal hernias that resulted in testicular atrophy. Indirect inguinal hernias are congenital, and although they can be repaired via herniotomy, adults who undergo this procedure have a high recurrence rate [8]. Since our cadaver showed significant testicular atrophy, it is reasonable to consider a parietal occipital variation of HFI as a possible differential. With the double indirect inguinal hernias, the origin of the testicular atrophy cannot be assumed. A medical file review and a comprehension history would be needed to correlate the atrophy to HFI over the hernias.

Clinically, HFI has been known to present alongside obesity and diabetes. Verdy et al. analyzed the correlation between HFI and higher body weight. This study of women aged 60 to 80 found that obese women have HFI at a prevalence of 84% compared to 16% in non-obese women [9]. Evidence of a correlation between HFI and metabolic disorders dates back to 3000 BC. In 2011, a group of scientists analyzed skeletal remains from a tomb underneath a royal palace of the Middle Bronze Age city of Qatna, Syria [10]. Individuals with a higher incidence of HFI were found buried with valuable items within a particular area of the tomb. Based on the archeological evidence, researchers believe these individuals were of a higher social status, and a correlation may exist between HFI and socioeconomic status. These individuals were more likely to have a high-calorie diet and sedentary lifestyle and were therefore more likely to have had metabolic disturbances such as diabetes mellitus and obesity [10]. Our specimen supports the conclusions of these studies because he was overweight. The body weight of our cadaver correlates to prior studies on HFI and, therefore, further solidifies this HFI variation as a differential.

Other syndromes associated with HFI include MSM and Troell-Junet syndromes. MSM syndrome is associated almost entirely with elderly females and is characterized by HFI, hormonal imbalances, neuropsychiatric diseases, and metabolic disorders such as obesity and diabetes [11]. The hormonal disturbances observed in MSM include hirsutism [11]. Interestingly, hirsutism is caused by an overproduction of androgens in females [12]. Since HFI in males is heavily associated with androgen deficiency, it has led some researchers to conclude that the functional disturbances of the gonads are a potential cause of hyperostosis [13]. Patients with MSM also often have severe headaches and psychotic symptoms [11]. It is important to note that these conclusions on MSM criteria result from case reports, and little formal research has been conducted on the syndrome. Troell-Junet syndrome is characterized by HFI, acromegaly, toxic goiter, and diabetes mellitus, indicating another potential correlation between hyperostosis and hormonal disturbances [14]. MSM and Troell-Junet syndrome both consist of generalized HFI symptoms and hormonal imbalances. Our specimen demonstrated hormonal disturbances with his testicular atrophy, leading us to consider MSM as a likely explanation for his condition. Hormonal studies and a glucose test would have been necessary to consider Troell-Junet as the preferred syndromic differential.

## Conclusions

HFI and HCI are usually undiagnosed and undiscovered unless the patient undergoes radiography. Deductions from the cadaveric dissection showed some major characteristics of HCI and HFI with interesting variations and features indicating MSM and Troell-Junet syndromes. Due to the lack of patient history and medical records, no further conclusions about clinical differentials, symptoms, or causative syndromes could be drawn. This report shows that further research needs to be conducted on HCI, HFI, and related syndromes to understand their presentations better.
